# Brain Responses to a 6-Hz Binaural Beat: Effects on General Theta Rhythm and Frontal Midline Theta Activity

**DOI:** 10.3389/fnins.2017.00365

**Published:** 2017-06-28

**Authors:** Nantawachara Jirakittayakorn, Yodchanan Wongsawat

**Affiliations:** Brain-Computer Interface Laboratory, Department of Biomedical Engineering, Faculty of Engineering, Mahidol UniversityNakhorn Pathom, Thailand

**Keywords:** EEG, binaural beat, brain response, dichotic stimulation, meditation

## Abstract

A binaural beat is a beat phenomenon that is generated by the dichotic presentation of two almost equivalent pure tones but with slightly different frequencies. The brain responses to binaural beats remain controversial; therefore, the aim of this study was to investigate theta activity responses to a binaural beat by controlling factors affecting localization, including beat frequency, carrier tone frequency, exposure duration, and recording procedure. Exposure to a 6-Hz binaural beat on a 250 Hz carrier tone for 30 min was utilized in this study. Quantitative electroencephalography (QEEG) was utilized as the recording modality. *Twenty-eight* participants were divided into experimental and control groups. Emotional states were evaluated by Brunel Mood Scale (BRMUS) before and after exposing to the stimulus. The results showed that theta activity was induced in the entire cortex within 10 min of exposure to the stimulus in the experimental group. Compared to the control group, theta activity was also induced at the frontal and parietal-central regions, which included the Fz position, and left hemisphere dominance was presented for other exposure durations. The pattern recorded for 10 min of exposure appeared to be brain functions of a meditative state. Moreover, tension factor of BRUMS was decreased in experimental group compared to control group which resembled the meditation effect. Thus, a 6-Hz binaural beat on a 250 Hz carrier tone was suggested as a stimulus for inducing a meditative state.

## Introduction

**Figure 1:**
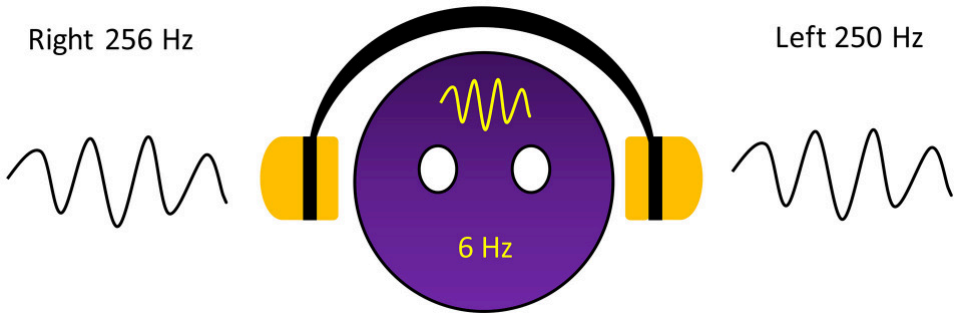
Six hertz binaural beat on a 250 Hz carrier tone generated within the brain.

The dichotic presentation of two almost equivalent pure tones but with slightly different frequencies leads to perceive fluctuations sounds which occurs as amplitude modulation generated by the two pure tones, or so-called beat, in the brain. The beat in this phenomenon is generated within the brain and is referred to as a binaural beat. The superior olivary complex is believed to be the first nucleus that receives auditory information from both sides of the ears, and binaurally activated phase-sensitive neurons are also found in the inferior colliculus (Kuwada et al., 1979; McAlpine et al., 1996, 1998; Spitzer and Semple, 1998; Schwarz and Taylor, 2005; Karino et al., 2006). The fluctuation in frequency equals the difference in the two pure tones that are presented. However, a classic study reported that the maximum difference in the two tones for which humans can perceive them as beat is 35 Hz; otherwise, 2 separate pure tones were perceived instead (Oster, 1973). For example, when a sinusoidal pure tone of 250 Hz is presented to the left ear and a 256 Hz is simultaneously presented to the right ear, amplitude modulation with a frequency rate of 6 Hz is perceived by the brain (Figure 1). In addition to the difference in the 2 tones, the carrier tone, which is the lower tone of the 2 tones, is also involved in the beat perception. One study measured perception of the beat on different frequency carrier tones and suggested that an intermediate frequency carrier tone of ~440 Hz facilitated the widest range of beat perception compared to lower and higher frequency carrier tones, which facilitated a narrower range (Licklider et al., 1950). The binaural beat induces an interesting effect termed the frequency following effect (Moushegian et al., 1973). This effect can induce brain activity corresponding to the perceived beat. The process of brain activity synchronization to the perceived beat is called entrainment (Wahbeh et al., 2007). Several studies have been conducted to investigate this effect and have attempted to determine the brain positions entrained by binaural beats.

An EEG experiment to investigate the brain responses to a 40-Hz binaural beat with low and high carrier tones of 400 Hz and 3,200 Hz, respectively, for 1.2 s was conducted. The results showed that 40 Hz activity is evoked, and the lower carrier tone induced higher responses than the higher carrier tone at the fronto-central region (Schwarz and Taylor, 2005). Other EEG studies have reported different results. Presentation of 7- and 15-Hz binaural beats to participants for 15 min led delta power to increase at the left temporal region for the 7-Hz binaural beat condition while gamma power increased for the 15-Hz condition (Lavallee et al., 2011). Utilization of 3- and 6-Hz binaural beats on 250 and 1,000 Hz carrier tones for 2 s. ERP responses were evoked at the left temporal region. The responses were higher for the 250 Hz condition than the 1,000 Hz condition and higher for 3-Hz than for 6-Hz (Pratt et al., 2009). These results were also reported in their next study (Pratt et al., 2010). Recording of EEG signal at only T3 and T4 positions while delivering 10- and 20-Hz binaural beats on a mean 400 Hz carrier tone to participants for 1 min was conducted. No change in alpha activity was detected in the 10-Hz condition, while the left temporal region expressed a higher amplitude of beta activity than the right side in the 20-Hz condition; however, clear evidence of a frequency following effect did not appear due to the number of recording electrodes (Vernon et al., 2012). A magnetic field study noted that the right temporal region responded to a 40-Hz binaural beat on a 500 Hz carrier tone within 1 s of exposure (Draganova et al., 2008). Other magnetoencephalography (MEG) studies also reported similar findings. A 26 Hz auditory steady-state response (ASSR) occurred at the right parietal and left middle frontal regions by exposure to a 26-Hz binaural beat on a 250 Hz carrier tone for 500 ms was reported (Chakalov et al., 2014). One study showed symmetrical responses to 4 binaural beats corresponding to 4.00- and 6.66-Hz beats on 240 and 480 Hz carrier tones for 10 min. ASSRs were induced at the temporal, frontal, and parietal regions, but symmetry did not always occur. However, these finding also suggested that the cerebral cortex can be synchronized with binaural beats (Karino et al., 2006). The brain responses to binaural beats by sweeping a beat for 16 s from 3- to 60-Hz on a carrier tone with a mean frequency of 500 Hz and found different polarities between the left and right auditory cortices. These researchers also suggested that different brain processes occur for different frequency activities (Ross et al., 2014). According to these findings, the responses of the brain to binaural beats remain debated; however, binaural beats can have behavioral effects, as suggested in several studies (Lane et al., 1998; Padmanabhan et al., 2005; Lavallee et al., 2011; Reedijk et al., 2013).

Several studies in literature have investigated different brain activities but one activity interested in nowadays lifestyle is theta activity and it is found during meditation. Theta activity is a type of brain activity classified by the frequency range of 4–8 Hz. It is associated with the behavioral states of alertness, attention, orientation, and working memory including the enhancement of cognitive and perceptual performances (Aftanas and Golocheikine, 2001; Stern et al., 2001). Theta activity also indicates concentration, focused attention, and a meditative state, and general theta and frontal midline theta rhythms are observed during meditation (Takahashi et al., 2005; Lagopoulos et al., 2009).

Meditation is the mental activity associated with attaining a deeply restful but fully alert state and is believed to reduce stress, which commonly occurs during daily life. One study claimed that 30 min of meditation is enough for a beginner to reduce stress (Sharma, 2015). Peacefulness and the reduction of stress are important for improving brain functions, especially cortical brain functions (Hankey and Shetkar, 2016). Theta activity has been utilized to investigate the meditative state by both general theta and frontal midline theta rhythms (Takahashi et al., 2005; Lagopoulos et al., 2009). Theta activity, during meditative state, is found at frontal and parietal-central regions but is not found at posterior region. This phenomenon is general theta activity. Theta activity must be found at frontal midline cortical position, specifically Fz position, which is considered as meditative state; and this is frontal midline theta rhythms. However, modern lifestyles are often characterized as stressful and highly active, and thus, 30 min of meditation may not achieve a meditative state if one is concerned of their surroundings; in such cases, it may take longer than 30 min to achieve a deep meditative state. Interestingly, if 6-Hz binaural beat can enhance theta activity due to the frequency following effect and if such enhanced activity shows a similar pattern to a meditative state that can be induced within a short duration, binaural beats may have clear applications for meditation.

With methodological differences in the primary stimuli parameters, i.e., beat frequency, carrier tone frequency, duration of exposure, and recording procedure, shown in literatures have varied across previous studies. This leads to difficultly in comparing the brain responses to binaural beats and hinders valid discussions. In addition, to reduce stress in nowadays lifestyle meditation becomes an interesting procedure; if binaural beat can induce similar activity of meditative state, it can be used as stimulus for meditation induction. Therefore, this study aimed to investigate the responses of the brain to a 6-Hz binaural beat on a 250 Hz carrier tone using QEEG for 30 min of listening. The 250 Hz carrier tone is chosen for the purpose of fair comparison with the existing works. As previous studies have lacked QEEG recordings when evaluating the responses to binaural beats, we sought to use QEEG as a recording procedure in this study. The reasons for applying 6-Hz binaural beat are as follows: 6-Hz is the middle of theta activity, 4–8 Hz, so it can represent theta activity, and indicate more precisely that occurred responses are appeared due to the stimulus in theta range not in the others. Only theta activity was observed due to the frequency following effect of binaural beats, as a 6-Hz binaural beat is in the range of theta activity. It was hypothesized that the power of the theta activity would be enhanced after listening to a 6-Hz binaural beat. Furthermore, binaural beats within theta activity have been utilized as stimuli in several studies, and thus, a 6-Hz binaural beat is selected for this investigation.

## Materials and methods

The aim of this study was to investigate the responses of the brain to a 6-Hz binaural beat on a 250 Hz carrier tone using QEEG with a 30-min listening period. Experimental and control groups were included. The experimental procedures involving human subjects described in this experiment were approved by the Institutional Review Board, Mahidol University with certificate of approval (COA) number 2015/074.1706.

### Experimental room

The experimental room was a sound-attenuated room with the temperature controlled to 25°C. The wall color of the room was white for neutral perceptions of emotion and mood, as reported by Sroykham et al. (2014). The experimental station was setup as follows: an armchair faced the white wall at a distance of 3 m, and the height of the armchair was adjusted to the comfort of each participant. A footrest was also placed in front of the armchair at a position that was comfortable for each participant.

### Binaural beat stimulus

The stimulus used in this study was a binaural beat stimulus that was specifically created for the experiment by providing two similar tones at slightly different frequencies. The carrier tone of 250 Hz was presented to the left ear, and the offset tone of 256 Hz was presented to the right ear. Using the Sound Forge Pro 11.0 program, the binaural beat stimulus was created, and the volume was set at 65 dB SPL.

### Participants

Twenty-eight participants with an average age of 21.9 years and a standard deviation of 1.9 years were included in the study. The participants were allocated to experimental and control groups. The purpose of the study and the experimental procedures were described to all participants before participation, but details of the stimulus were not revealed. The experimental group was composed of 17 participants, 5 females and 12 males, with average age of 22.2 years and a standard deviation of 2.1 years, while the control group was composed of 11 participants, 4 females and 6 males, with an average age of 21.4 years and a standard deviation of 1.5 years. The aims and procedures of the experiment were told to the participants. All participants were asked to give written informed consent before participation in the study. The participants were free to stop their participation during the experiment or to withdraw themselves from the experiment for any reason.

### EEG recording

Each participant was asked to sit in the armchair within the experimental room in an upright position and to lay their feet on the footrest and relax. The size of participant's head was measured for fitting a suitably sized electrode cap. The electrode cap, composed of an elastic cap with mesh electrodes at the positions corresponding to the international 10/20 system, was worn on the participant's head. Each electrode was filled with conductive gel to reduce the impedance between each electrode and the scalp to improve the recordings and to minimize noise. The impedance was <5 kΩ. Two electrode cups with conductive paste were then clipped on both the right and left ear lobules as ground and reference, respectively. All electrodes were wired to a BrainMaster® system for recording EEG signals using BrainMaster Discovery software. A Monster® Inspiration over-ear headphones were also worn by the participant after setting the electrodes.

### Experimental procedures

**Figure 2:**
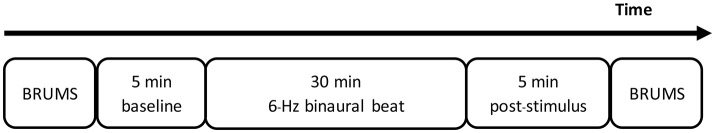
Experimental timeline.

After setting EEG apparatus, all participants underwent the experimental procedures which began with emotional states evaluation by BRMUS, continuous EEG recording for 40 min, and ended up with emotional states evaluation once again (Figure 2).

The BRMUS is a self-report emotional state questionnaire composed of 24 items. These items correspond to a 6-factor model including “Anger,” “Confusion,” “Depression,” “Fatigue,” “Tension,” and “Vigor.” Each item can be responded to according to the 5-point Likert scale, which ranges from 0 to 4 representing “not at all” to “extremely” depending on the participant's feelings.

Consequently, 40 min of the experimental period began. The experimental period included 5 min of baseline recordings, 30 min of stimulus period, and a final 5 min of post-stimulus recording. The stimulus was provided to each participant based on the participant's group. The stimulus of experimental group was a 6-Hz binaural beat while that of the control group was silence. However, all participants were told that the stimulus was already provided. Along the experimental period, EEG signals were recorded, and participants were asked to continue to sit idly and to make as few movements as possible. However, due to long experimental period, fatigue occurred in some participants. If the participants reported feelings of fatigue, a short duration of movements was permitted to release the fatigue. The signals recorded during movement and eye blinking were not included in the analysis. During this 40 min of recording, all participants were asked to focus on the stimulus but can randomly think without did any kind of the meditation.

After the 40-min experimental period, the participants were again evaluated via the BRUMS to evaluate their emotional states after the experiment.

### Data analysis

The recorded EEG signals included 40 min for each participant. Each recorded EEG signal was separated as follows: 5 min of baseline recording, followed by 6 intervals of 5 min each along the stimulus period, and finally 5 min of post-stimulus recording. A total of 8 intervals of 5 min each were recorded. The EEG signals of each 5-min interval were selected based on the following criteria: clear signals without noise, eye blinking, or artifacts, and split half and test retest reliabilities of at least 90%. Fast Fourier transformation (FFT) was performed on each selected signal to convert the signal from the time domain to the frequency domain. The absolute power of the theta activity was assessed for statistical analysis. All data analyses were conducted by NeuroGuide software. The reasons considering 5-min in each time-point were to provide uncomplicated comparison with literatures that investigate the responses in term of series of 5 min, i.e., 10, 15 min, etc., and 30 min was recommended that enough for beginner in meditation, also be series of 5 min (Sharma, 2015).

### Statistical analysis

Within the same group, paired *t*-tests were conducted to compare mean of absolute power of the theta activity between each interval and baseline, position-by-position. The comparisons were performed to indicate the time-point of increased FFT absolute power of theta activity upon listening to the stimulus for both the experimental and control groups. *P* < 0.05 were considered significant. In addition, paired *t*-tests were also conducted to compare the changes in the BRUMS scores before and after listening to the stimulus. However, *p* < 0.1 were considered different because the internal consistency of the BRUMS was reported as 0.90 (McNair et al., 1992).

Between the experimental and control groups, independent *t*-tests were conducted to compare the mean differences ($\overset{®}{d}$ in paired *t*-test) between the theta activity of each interval and baseline between the two groups, position-by-position. The changes in theta activity were compared to determine whether the changes that occurred at each time-point were a result of the stimulus rather than idly sitting. *P* < 0.05 were considered significant. The same statistical analyses were performed for the BRUMS scores to indicate the differences in emotional states between the 2 groups. *P* < 0.05 were also considered significant.

## Results

The stimuli were presented to participants for 30 min depending on participants' group. A 6-Hz binaural beat was presented to the experimental group, and silence was presented to the control group. The brain responses to the stimulus were investigated by comparing each exposure duration, comprising 5-min segments of the 30-min recording and including 5 min of post-stimulus and baseline.

### Theta activity after listening to a 6-Hz binaural beat for 30 min

#### Baseline recording

**Figure 3:**
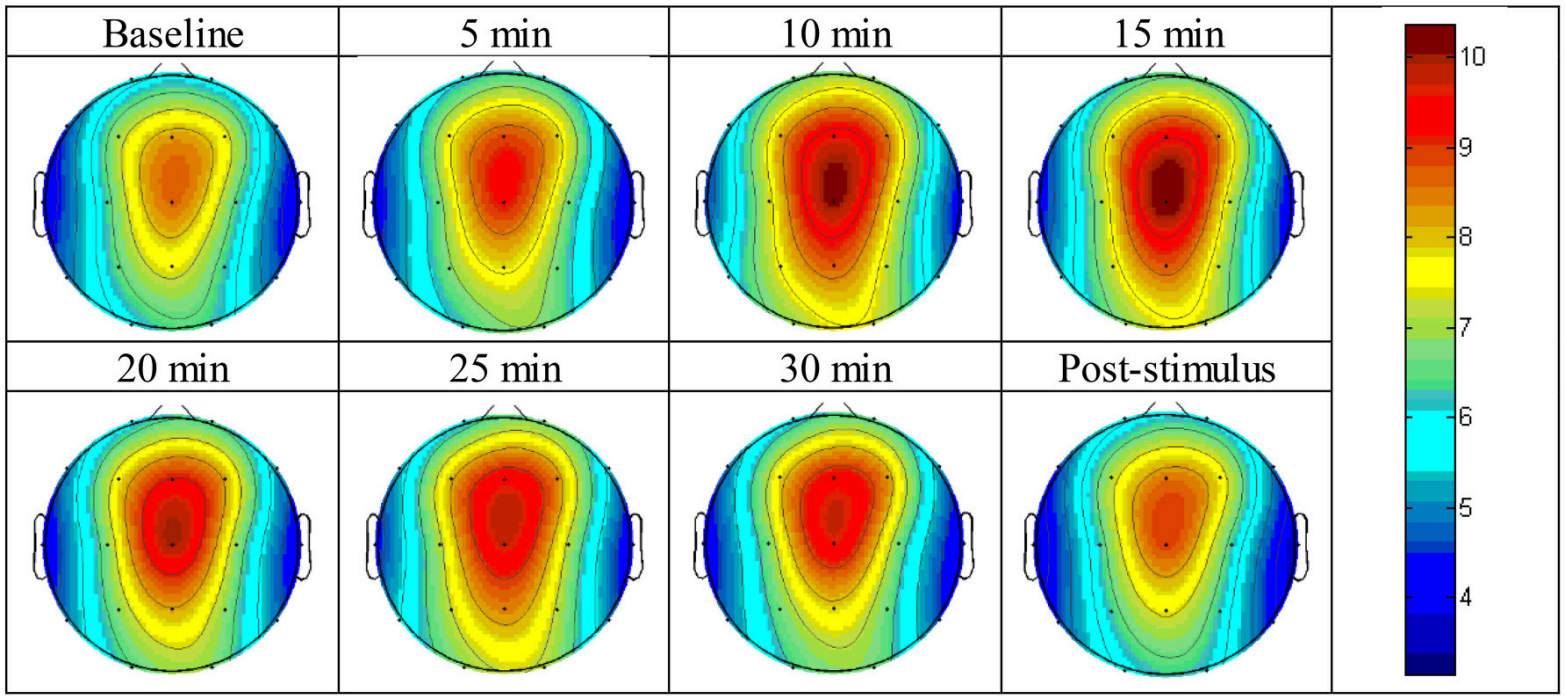
Brain topographic mapping of theta activity form baseline to the post-stimulus periods of the experimental group.

The absolute power of the theta activity showed a maximum value at the fronto-central cortical position, specifically between the Fz and Cz positions (Figure 3). The absolute power of the theta activity gradually declined from the maximum position in radial fashion and reached a minimum value at the temporal cortical positions on both sides, i.e., the T3 and T4 positions. The values are reported in Supplementary Figure 1.

#### During stimulus exposure

After listening to the 6-Hz binaural beat stimulus, the absolute power of the theta activity was enhanced at nearly all cortical positions except the T4 position within 10 min of exposure (Table 1). Paired *t*-test analyses of the absolute power of theta activity within 10 min compared to baseline are shown in Table 2. This time-point presented the most cortical positions that were significantly changed by the stimulus and the maximum changes in theta activity power compared to baseline.

**Table 1:** Cortical positions with significantly different absolute power of theta activity at each interval compared to baseline in the experimental group.

**Listening interval (min)**	**Electrode position**
5	N/A
10	Fp1, F3, C3, P3, O1, F7, T3, T5, Fz, Fp2, F4, C4, P4, O2, F8, T6, Cz, Pz
15	Fp1, F3, P3, O1, F7, Fz, Fp2, F4, P4, O2, Cz, Pz
20	F3, Fz, F4, Cz
25	F3, F7, Fz, Fp2, F4, P4, O2, Cz
30	Cz
Post-stimulus	N/A

**Table 2:** Paired *t*-test analysis of the absolute power of theta activity at 10 min compared to baseline in the experimental group (only significant differences are shown).

**Channel**	**10 min**	**Baseline**	**Paired differences**			***P*-value**
			**Mean difference**	**Std. deviation**	**Std. error mean**	
Fp1	6.4480	5.5575	0.8905	1.3969	0.3388	0.0091
F3	8.0915	6.9892	1.1023	1.7809	0.4319	0.0107
C3	7.3098	6.3205	0.9894	1.8143	0.4400	0.0195
P3	7.5529	6.4712	1.0818	1.6491	0.4000	0.0078
O1	6.9537	5.9747	0.9790	1.5079	0.3657	0.0083
F7	5.3288	4.5624	0.7665	1.2081	0.2930	0.0094
T3	4.4969	3.8970	0.5999	1.0899	0.2643	0.0187
T5	5.8147	4.9203	0.8944	1.6750	0.4062	0.0214
Fz	9.5459	8.2291	1.3167	2.1602	0.5239	0.0115
Fp2	6.7616	5.7019	1.0597	1.5947	0.3868	0.0073
F4	8.4720	7.4843	0.9877	1.9132	0.4640	0.0246
C4	7.2434	6.4475	0.7959	1.4541	0.3527	0.0192
P4	7.2147	6.2366	0.9781	1.4273	0.3462	0.0061
O2	7.3251	6.2407	1.0845	1.2904	0.3130	0.0016
F8	5.4432	4.9739	0.4693	0.9190	0.2229	0.0257
T6	5.2303	4.6753	0.5550	1.0731	0.2603	0.0244
Cz	10.1346	8.6357	1.4990	2.5943	0.6292	0.0150
Pz	8.7061	7.5102	1.1959	2.0133	0.4883	0.0131

Figure 3 displays the topographic mapping of theta activity for all intervals of the experiment and indicated that similar patterns of theta activity appeared at each time-point and that the maximum absolute power of theta activity was expressed at the fronto-central cortical position. The maximum power gradually declined in radial fashion. The minimum value was presented at the temporal cortical positions on both sides.

Table 1 shows the significantly different cortical positions of the absolute power of theta activity according to paired *t*-tests for each interval after exposure to the stimulus compared to baseline. The significant differences suggested that within 10 min of exposure, all cortical positions were enhanced by the stimulus; at 15 and 25 min, fewer cortical positions were changed by the stimulus. Increased theta activity was observed at the frontal and parietal-central regions, which is a pattern similar to a meditative state. All values of absolute power of theta activity for the paired *t*-tests are given in Supplementary Figure 1 and Supplementary Table 1.

#### Post-stimulus recording

Within the post-stimulus period, the absolute power of theta activity was not different from the baseline duration (Supplementary Figure 1). No significant difference was found based on a paired *t*-test.

### Theta activity after sitting for 30 min

#### Baseline recording

**Figure 4:**
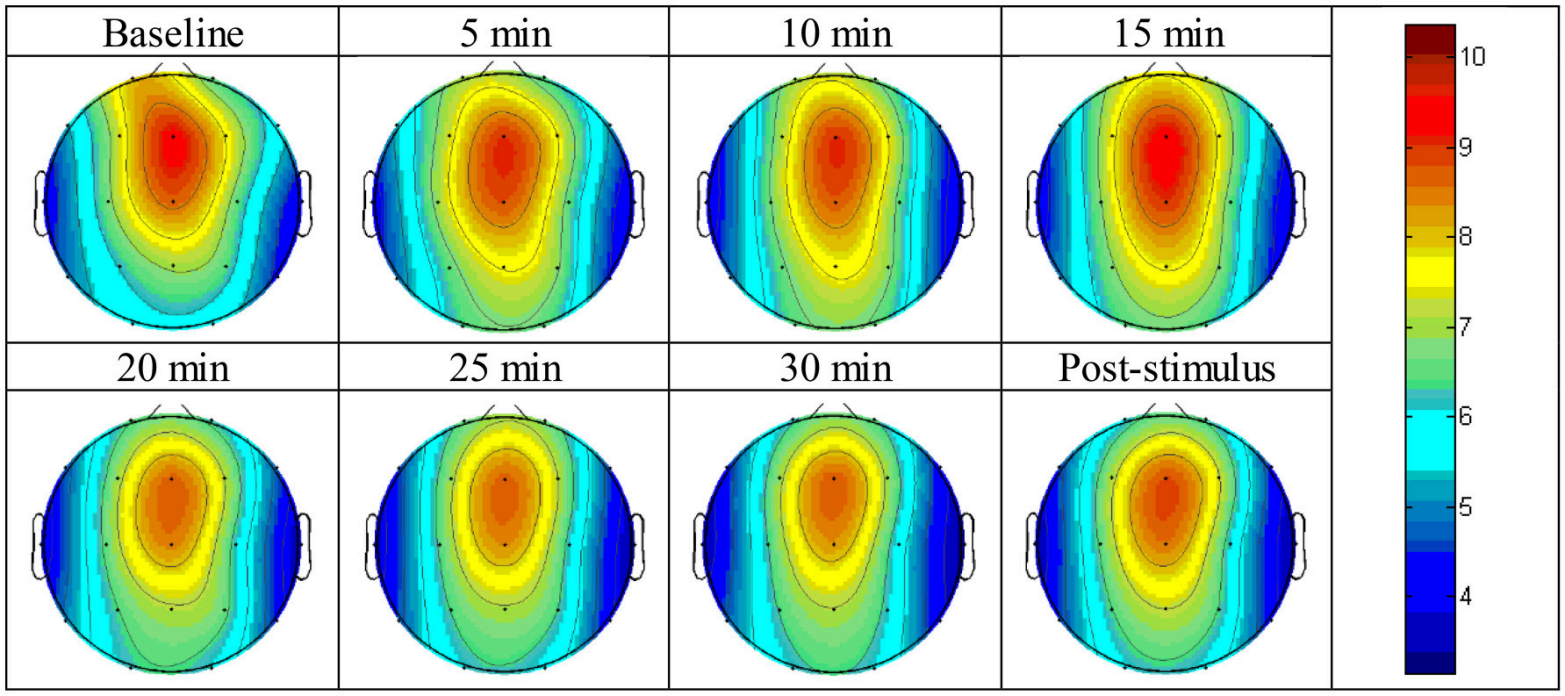
Brain topographic mapping of theta activity form baseline to the post-stimulus periods of the control group.

The absolute power of theta activity was maximum at the fronto-central cortical position, specifically between the Fz and Cz positions (Figure 4). A similar pattern as that observed in the experimental group occurred: the absolute power of theta activity gradually reduced from the maximum position in radial fashion, and the minimum power was observed at the temporal cortical positions on both sides, i.e., the T3 and T4 positions. However, the Fp1 position showed a value near the maximum value. The values of absolute power of theta activity are displayed in Supplementary Figure 2.

#### During stimulus exposure

The absolute power of theta activity increased at the O1 and O2 positions within 5 min of idly sitting and listening to silence during the 30-min control period. However, no significant difference was found within 10 min of exposure (Table 3).

**Table 3:** Cortical positions with significantly different absolute power of theta activity at each interval compared to baseline in the control group.

**Listening interval (min)**	**Electrode position**
5	O1, O2
10	N/A
15	O1
20	N/A
25	N/A
30	N/A
Post-stimulus	N/A

Figure 4 shows the topographic mapping of theta activity for all intervals of the experiment indicating that a similar pattern of theta activity appeared at each time-point, although the absolute power decreased with time.

Table 3 shows the significantly different cortical positions of the absolute power of theta activity according to paired *t*-tests for each interval after exposure to the stimulus compared to baseline. The paired *t*-tests results did not reveal any significance within the rest intervals except at the 5-min time-point and 15-min time-point. All value of the absolute power of theta activity for the paired *t*-test are given in Supplementary Figure 2 and Supplementary Table 2.

#### Post-stimulus recording

Within the post-stimulus period, the absolute power of theta activity decreased compared to baseline (Supplementary Figure 1) but no significance was found according to paired *t*-tests.

### Comparison of the changed theta activity between experimental and control groups

To investigate whether a 6-Hz binaural beat could induce theta activity in experimental group, independent *t*-tests were conducted to compare experimental and control groups position-by-position based on the mean difference at each time-point and comparing to the baseline of both groups. Analyses were performed only for significantly different positions in the experimental group.

Table 4 shows the independent *t*-tests of the mean difference in theta activity at the 10-min time-point. Significant difference appeared at the frontal and parietal-central regions. The significant differences suggested that theta activity was enhanced by the 6-Hz binaural beat because significance was found within the experimental group over baseline, indicated by the mean difference ($\overset{®}{d}$), and the changes at the same time-points compared to the control group were also significantly different. In other words, the significance of $\overset{®}{d}$ indicated that increases in theta activity of the experimental group due to the 6-Hz binaural beat were a result of the stimulus rather than idly sitting.

**Table 4:** Independent *t*-test analysis of the mean differences ($\overset{®}{d}$) in the absolute power of theta activity between the experimental and control groups at 10 min compared to baseline.

**Channel**	$\overset{®}{d}$ **of theta activity**		***SD*** **of** $\overset{®}{d}$		***P*-values**	**Sig**.
	**Experimental**	**Control**	**Experimental**	**Control**		
Fp1	0.8905	−1.0075	1.3969	3.5553	0.0628	
F3	1.1023	−0.2477	1.7809	1.0678	0.0163	^*^
C3	0.9894	−0.0502	1.8143	0.9044	0.0236	^*^
P3	1.0818	0.4047	1.6491	1.2610	0.1288	
O1	0.9790	0.7079	1.5079	1.3923	0.3182	
F7	0.7665	−0.5507	1.2081	1.1070	0.0037	^*^
T3	0.5999	−0.2380	1.0899	0.6599	0.0154	^*^
T5	0.8944	0.0310	1.6750	0.8558	0.0377	^*^
Fz	1.3167	−0.2475	2.1602	2.1060	0.0350	^*^
Fp2	1.0597	0.2346	1.5947	1.3328	0.0834	
F4	0.9877	−0.1702	1.9132	1.7751	0.0600	
C4	0.7959	−0.0649	1.4541	1.2460	0.0592	
P4	0.9781	0.4113	1.4273	1.5897	0.1676	
O2	1.0845	0.8134	1.2904	1.8493	0.3254	
F8	0.4693	−1.0251	0.9190	1.7623	0.0063	^*^
T4	0.3348	−0.3325	0.8714	1.2282	0.0520	
T6	0.5550	0.1935	1.0731	1.4419	0.2268	
Cz	1.4990	0.0353	2.5943	1.3537	0.0284	^*^
Pz	1.1959	0.6325	2.0133	1.8484	0.2312	

* Indicate significant difference of theta activity at 10-minute time-point. The meaning of “^*^” in the table is increase in theta activity of experimental group at significant position being a result of 6-Hz binaural beat stimulus not a result of idle sitting.

Table 5 shows the cortical positions with significantly different mean differences in theta activity compared to the same time-points in the control group, which appeared at 20 and 25 min at the frontal region. All time-points significantly involved the fronto-central cortical position or the Fz position which, is indicated as frontal midline theta activity. Mean differences in the values of both groups at each time-point compared to baseline are shown in Supplementary Figure 3, and the standard deviations of both groups at each time-point compared to baseline are shown in Supplementary Tables 3, 4, respectively, for the experimental and control groups.

**Table 5:** Cortical positions with significantly different mean differences ($\overset{®}{d}$) in the absolute power of theta activity at each interval between the experimental and control groups (independent *t*-tests were conducted for significant channels found in the experimental group).

**Listening interval (min)**	**Electrode position**
5	N/A
10	F3, C3, F7, T3, T5, Fz, F8, Cz
15	N/A
20	F3, Fz, F4, Cz
25	F3, F7, Fz, F4
30	N/A
Post-stimulus	N/A

### Emotional states after listening to the 6-Hz binaural beat for 30 min

The emotional states of the participants in the experimental group changed after the experimental procedures had finished and were given in Table 6. Paired *t*-tests indicated that anger, depression, fatigue, and confusion were significantly increased, while tension and vigor were significantly decreased compared to baseline.

**Table 6:** Average BRUMS scores for 24 items before and after listening to 30 min of the 6-Hz binaural beat for the experimental group with the significant increase (up arrows) and decrease (down arrows) *SD* indicates the standard deviation of the difference between before and after listenin ($\overset{®}{d}$).

**Factors**	**Items**	**Before**	**After**	***SD***	**Significance**
Tension	Panicky	0.1765	0.2941	0.6002	
	Anxious	0.4118	0.5294	0.4851	
	Worried	0.7059	0.4118	0.5879	↓↓
	Nervous	0.6471	0.6471	0.6124	
Anger	Annoyed	0.4118	1.1765	1.0326	↑↑
	Bitter	0.3529	0.5294	0.8090	
	Angry	0.1765	0.1765	0.7071	
	Bad tempered	0.1765	0.3529	0.3930	↑↑
Depression	Depressed	0.2353	0.4118	0.3930	↑↑
	Downhearted	0.2353	0.2941	0.4287	
	Unhappy	0.4706	0.7059	0.8314	
	Miserable	0.1765	0.1176	0.2425	
Fatigue	Worn out	0.7059	1.1765	1.1789	↑
	Exhausted	1.0588	1.7059	1.1695	↑↑
	Sleepy	1.2941	3.1765	1.4527	↑↑
	Tired	1.2353	1.7059	1.1789	↑
Vigor	Lively	1.4706	0.5882	0.7812	↓↓
	Energetic	1.0588	0.5882	0.9432	↓↓
	Active	1.1176	0.5294	0.7952	↓↓
	Alert	1.2941	0.7059	0.8703	↓↓
Confusion	Confused	0.8235	1.1765	0.8618	↑
	Muddled	0.5882	0.8235	0.9701	
	Mixed up	0.7059	1.0000	0.7717	↑
	Uncertain	0.4118	0.4118	0.3536	

### Emotional states after sitting for 30 min

The emotional states of the participants in the control group changed after the experimental procedures had finished and are given in Table 7. Paired *t*-tests indicated that anger and fatigue significantly increased, while vigor significantly decreased compared to baseline. The rest of the factors, including tension, depression, and confusion were not significantly different.

**Table 7:** Average BRUMS scores for 24 items before and after listening to 30 min of the stimulus for control group with the significant increase (up arrows) and decrease (down arrows) *SD* indicates the standard deviation of the difference between before and after listenin ($\overset{®}{d}$).

**Factors**	**Items**	**Before**	**After**	***SD***	**Significance**
Tension	Panicky	0.4545	0.4545	1.3416	
	Anxious	0.8182	0.6364	0.9816	
	Worried	0.6364	0.4545	0.6030	
	Nervous	0.9091	1.0000	1.2210	
Anger	Annoyed	0.3636	0.8182	0.5222	↑↑
	Bitter	0.4545	0.8182	1.5015	
	Angry	0.0000	0.0000	0.0000	
	Bad tempered	0.2727	0.4545	0.6030	
Depression	Depressed	0.0909	0.4545	0.9244	
	Downhearted	0.1818	0.4545	1.1909	
	Unhappy	0.5455	0.5455	0.6325	
	Miserable	0.2727	0.6364	0.9244	
Fatigue	Worn out	0.7273	1.5455	1.0787	↑↑
	Exhausted	1.8182	2.4545	1.0269	↑↑
	Sleepy	1.1818	2.4545	1.3484	↑↑
	Tired	1.2727	1.9091	1.0269	↑↑
Vigor	Lively	2.1818	1.0909	0.8312	↓↓
	Energetic	1.4545	0.6364	1.4709	↓↓
	Active	1.3636	0.5455	1.5374	↓
	Alert	1.2727	0.7273	1.0357	↓
Confusion	Confused	0.8182	0.9091	1.0445	
	Muddled	1.0000	1.0000	1.0000	
	Mixed up	0.9091	0.8182	1.5136	
	Uncertain	0.2727	0.6364	1.0269	

### Comparison of the changed emotional states between experimental and control groups

The “worried” item, which is one item of the tension factor, was significantly lower after the experimental procedures had finished only in the experimental group; therefore, worried feelings decreased due to the 6-Hz binaural beat. Similar to “worried,” the “bad tempered” item, which is one item of the anger factor, “depressed,” which is one item of the depression factor, and “confused” and “mixed up,” which are two items of the confusion factor, were all significantly higher in the experimental group; therefore, these feelings increased due to the 6-Hz binaural beat.

The rest of the items that were significantly changed in both groups were compared to indicate whether the changes that occurred in the experimental group differed from those in the control group. Independent *t*-tests were conducted on the mean difference ($\overset{®}{d}$) of the BRUMS ranges between both groups for items that correspond to significant differences in both groups. The results are presented in Table 8. None of the states showed a significant difference, indicating that these emotional states did not differ between groups.

**Table 8:** Independent *t*-test analysis of the mean difference ($\overset{®}{d}$) of the BRUMS ranges between the experimental and control groups.

	**Item**	$\overset{®}{d}$ **of BRUMS ranges**		***SD*** **of** $\overset{®}{d}$		***P*-value**	**Sig**.
		**Experimental**	**Control**	**Experimental**	**Control**		
Increased	Annoyed	0.7647	0.4545	1.0326	0.5222	0.3427	
	Worn out	0.4706	0.8182	1.1789	1.0787	0.4630	
	Exhausted	0.6471	0.6364	1.1695	1.0269	0.9815	
	Sleepy	1.8824	1.2727	1.4527	1.3484	0.3015	
	Tired	0.4706	0.6364	1.1789	1.0269	0.7207	
Decreased	Lively	−0.8824	−1.0909	0.7812	0.8312	0.5300	
	Energetic	−0.4706	−0.8182	0.9432	1.4709	0.4772	
	Active	−0.5882	−0.8182	0.7952	1.5374	0.6803	
	Alert	−0.5882	−0.5455	0.8703	1.0357	0.9121	

## Discussion

Many investigations of brain responses to binaural beat stimuli have been conducted; however, the results are controversial and continue to be debated. Several factors including beat frequency, carrier tone frequency, exposure duration, and recording procedures interfere with the discussion process, as these factors affect brain responses, and differences in these factors do not allow for clear comparisons among studies. This study attempted to determine the responses by controlling for all factors except exposure duration by utilizing quantitative power. The stimulus utilized in this study was a 6-Hz binaural beat on a 250 Hz carrier tone. Theta activity corresponding to the received beat was investigated, as 6-Hz is in the range of theta activity, based on the hypothesis that theta activity can be entrained by the stimulus and that such entrainment is a dynamic process related to exposure duration.

The results suggested that theta activity was entrained by the 6-Hz binaural beat. Upon listening to the 6-Hz binaural beat for 10 min, theta activity was entrained at almost all cortical positions in the experimental group. This entrainment was not found in the control group, which listened to silence and idly sat in the experimental room. Interestingly, both groups underwent the same procedures, differing only in stimulus exposure; therefore, to elucidate the effect of idly sitting, which can generate theta activity because this activity is also common during meditation, the entrained theta activity between the experimental group and the control group was compared. The results indicated that increases in theta activity of the experimental group resulted from the 6-Hz binaural beat but not idly sitting. These effects seemingly occurred following 20 and 25 min of exposure, although different cortical positions expressed the entrainment. Significantly affected cortical positions included the frontal and parietal-central regions at all time-points, especially at 10 min of exposure; however, at 20 and 25 min, significance was primarily observed at the frontal region. This evidence supported the hypothesis that the brain responses to binaural beat were dynamic processes due to the exposure duration, the results revealed different entrainments of cortical positions for different exposure durations. Furthermore, the theta activity entrained by the 6-Hz binaural beat in this study also exhibited a frequency following effect of binaural beat. Another interesting point is the localization of significant differences revealed by the statistical analysis. At all time-points, the left hemisphere presented more dominant significance than the right hemisphere. This apparent localization is in line with several previous studies that have recorded EEG signals. It should be noted that comparison of the responses in this study to previous studies cannot provide directly but only the trend of responses because of EEG reference factor. In this study, left ear was utilized as reference for all EEG channels due to specification of the hardware utilized for EEG recording. Reference choices was reported to have some effects on spatio-temporal analysis of the brain activity (Yao et al., 2007) but in this study, the comparisons were conducted within the same participant at different time-points thus, common errors were eliminated. Further, comparison in cross-study can be applied by reference electrode standardization technique (REST, Yao, 2001; Yao et al., 2005).

The dynamic process of the brain responses to the binaural beat also suggested the suitable exposure duration for 6-Hz binaural beat. Although, 10, 20, and 25 min all corresponded to significant differences, different absolute powers were found. At 10 min, the absolute power of theta activity entrained by the 6-Hz binaural beat was maximum among the resting time-points of which lower values were presented (Supplementary Figure 1). Therefore, the suitable exposure duration was 10 min. For this duration of stimulation, the maximum absolute power of theta activity was entrained at all cortical regions. Listening to the 6-Hz binaural beat for more than 10 min did not induce higher power of theta activity, and furthermore, fatigue may occur.

Idle sitting is similar to meditation; therefore, the theta activity pattern of medication state could be exhibited by participants, but it did not appear. The results showed that the absolute power of theta activity at all cortical positions in the control group decreased with the time of idle sitting (Supplementary Figure 2), which suggests opposition to meditation. The results of the control group verified that the absolute power of theta activity in the experimental group was entrained by the 6-Hz binaural beat.

During meditation, theta activity of the meditative state presents as a general theta rhythm and frontal midline theta activity (Takahashi et al., 2005; Lagopoulos et al., 2009). In general theta rhythm, the power of theta activity is higher than normal at the frontal and parietal-central regions but does not appear at posterior regions. In frontal midline theta activity (FmTheta), the power of theta activity is higher at the frontal midline cortical position, specifically at the Fz position, and without this activity, a meditative state is not achieved. Interestingly, the absolute power of theta activity at 10 min of stimulus exposure in the experimental group exhibited both general theta rhythm at the frontal and parietal-central regions and frontal midline theta activity at the Fz cortical position. Moreover, compared to the control group, these patterns of theta activity were also presented at 10 min of exposure; however, frontal midline theta activity was also presented at other time-points. This finding appears to indicate that the 6-Hz binaural beat could be utilized to induce a meditative state within a short duration.

For evaluation of emotional states by the BRUMS questionnaire, several emotional states in the 6-Hz binaural beat condition differed from the control condition, including “worried,” “bad tempered,” “depressed,” “confused,” and “mixed up.” The “worried” state was decreased by the 6-Hz binaural beat condition, while the other emotional states were increased. These emotional states were changed due to the stimulus, as they were not observed in the control group. Possible explanations of these changes could be as follows: the 6-Hz binaural beat can reduce “worried” feelings; however, “bad tempered,” “depressed,” “confused,” and “mixed up” may be a result of over-stimulation. The rest of the emotional states evaluated by the BRUMS did not correspond to any difference between the experimental and control groups (Table 8). The lack of differences may be due to the fact that the experimental procedures of both the experimental and control groups included idly sitting for 40 min, and the emotional states were evaluated by the BRUMS to indicate the emotions of the participants after the experiment had finished. However, all items of the “vigor” factor decreased, and all items of the “fatigue” factor increased in both groups, which might reflect either over-stimulation or the long period of sitting idly. This lack of differences between experimental and control groups indicates that sitting idly is different from meditation. Normally, “annoyed” feelings, which is negative emotion, is potentially eliminated during meditation; and “worn out,” “exhausted,” “sleepy,” and “tired” do not appear during meditation. Moreover, decreases in being powerful and energetic are also disappear because meditation induces peacefulness, claim, and reduces stress with full consciousness. Both theta activity and emotional states responses seem to be enough evidences for differentiating between meditation and sitting idly, and indicating that theta activity explaining in this study is due to the stimulus; in addition, “worried” feelings reduce also be effect of the stimulus. Therefore, a suitable exposure duration of 10 min for inducing a 6-Hz binaural beat is suggested.

## Limitation

This study investigated only the brain responses to a 6-Hz binaural beat on a 250 Hz carrier tone for only a single exposure, although habituation or adaptive brain responses from several exposures should be further investigated. The brain responses to different binaural beat stimuli should be provided, as different brain responses occur due to different beat frequencies corresponding to different brain activities. Finally, the emotional evaluation in this study was performed over the same period, and therefore, emotional evaluations must be conducted at suitable time-points to investigate emotional states due to the 6-Hz binaural beat for utilizing 6-Hz binaural beats in alternative applications.

### Suggested applications

The brain responses to the 6-Hz binaural beat revealed in this study showed that theta activity was enhanced similar to that observed in a meditative state within just 10 min of exposure. Therefore, a 6-Hz binaural beat on a 250 Hz carrier tone could be utilized as a stimulus for inducing a meditative state, as meditation has several advantages, such as stress reduction. However, achieving such effects via meditation require a deep meditative state that often takes a long period of time. With this binaural beat, a meditative state can be induced in just 10 min and may be more feasibly implemented in modern lifestyles.

## Conclusion

Theta activity was induced by a 6-Hz binaural beat on a 250 Hz carrier tone. Responses occurred at all cortical regions; however, compared to the control group, the induced responses at the frontal and parietal-central regions were left hemisphere dominant. Moreover, the pattern of theta activity was similar to that of a meditative state, in which general theta rhythms were increased at the frontal and parietal-central regions and frontal midline theta activity appeared at the Fz position within 10 min stimulus exposure. Therefore, we suggest that a 6-Hz binaural beat on a 250 Hz carrier tone could be used as a stimulus for inducing a meditative state within a short duration.

## Author contributions

NJ designed and conducted experimental protocol. YW verified experimental protocol.

### Conflict of interest statement

The authors declare that the research was conducted in the absence of any commercial or financial relationships that could be construed as a potential conflict of interest.
