# Supplementary material for: Brain Responses to a 6-Hz Binaural Beat: Effects on General Theta Rhythm and Frontal Midline Theta Activity
Source: Front Neurosci. 2017 Jun 28;11:365. doi: 10.3389/fnins.2017.00365 (PMC5487409; doi:10.3389/fnins.2017.00365)
Supplement: Supplementary file 1 [file DataSheet1.docx]

Supplementary Fig. 1 Average absolute power of theta activity of all electrodes in each interval for the experimental group.

Supplementary Fig. 2 Average absolute power of theta activity of all electrodes in each interval for the control group.

Supplementary Fig. 3 The mean differences ($\bar{d}$) of theta activity before and after listening to the stimulus for the experimental and control groups in each interval.

(A) After 5 min – baseline. (B) After 10 min – baseline.

(C) After 15 min – baseline. (D) After 20 min – baseline.

(E) After 25 min – baseline. (F) After 30 min – baseline.

(G) Post-stimulus – baseline.

Supplementary Table 1. Standard deviations of absolute power of theta activity at all intervals for the experimental group.

| **Duration**  **Channel** | **Baseline** | **5**  **minutes** | **10**  **minutes** | **15**  **minutes** | **20**  **minutes** | **25**  **minutes** | **30**  **minutes** | **Post-**  **stimulus** |
| --- | --- | --- | --- | --- | --- | --- | --- | --- |
| Fp1 | 1.9173 | 3.2206 | 2.9307 | 2.2710 | 2.1816 | 2.5046 | 2.4148 | 1.7333 |
| F3 | 2.3287 | 3.9324 | 3.5270 | 2.7269 | 2.6420 | 3.4655 | 3.5806 | 2.5382 |
| C3 | 2.0635 | 3.1064 | 2.9649 | 2.3598 | 2.0994 | 2.6199 | 2.6994 | 2.1111 |
| P3 | 2.5066 | 3.6761 | 3.2401 | 2.9601 | 2.1369 | 2.9978 | 2.5869 | 2.1349 |
| O1 | 2.2859 | 3.2799 | 3.1704 | 3.1114 | 2.0871 | 3.2207 | 2.8355 | 2.0797 |
| F7 | 1.0822 | 1.9537 | 2.0186 | 1.4183 | 1.3822 | 1.9115 | 1.8091 | 1.2165 |
| T3 | 1.0092 | 1.5960 | 1.9111 | 1.3581 | 1.2474 | 1.7083 | 1.7054 | 1.2144 |
| T5 | 1.8890 | 2.3400 | 2.9606 | 2.1775 | 1.6617 | 2.5494 | 2.0205 | 1.6192 |
| Fz | 2.7574 | 4.7357 | 3.9402 | 3.5167 | 3.0003 | 4.0466 | 4.0338 | 2.8524 |
| Fp2 | 1.9780 | 3.4398 | 2.9893 | 2.5124 | 2.5052 | 3.0651 | 2.8515 | 2.0934 |
| F4 | 2.9579 | 4.4334 | 3.8706 | 3.3303 | 2.8783 | 3.8058 | 3.8923 | 2.8925 |
| C4 | 2.3385 | 3.5008 | 3.0404 | 2.8374 | 2.2373 | 2.9173 | 2.8742 | 2.1969 |
| P4 | 2.3114 | 3.6828 | 3.1600 | 3.0022 | 2.2769 | 3.1804 | 2.7008 | 2.1106 |
| O2 | 3.0792 | 4.3038 | 3.5968 | 3.8205 | 2.6676 | 3.7757 | 3.2475 | 2.2271 |
| F8 | 1.7565 | 2.4637 | 2.1190 | 2.0132 | 1.9981 | 2.2391 | 2.1686 | 1.4913 |
| T4 | 1.5217 | 1.8908 | 1.8049 | 1.6646 | 1.3821 | 1.7270 | 1.6602 | 1.2013 |
| T6 | 1.9374 | 2.4510 | 2.3217 | 2.1012 | 1.8530 | 2.2933 | 1.9287 | 1.5020 |
| Cz | 2.8177 | 4.7336 | 4.1639 | 4.2859 | 3.4528 | 3.5807 | 3.8883 | 3.1806 |
| Pz | 2.7709 | 4.1689 | 3.7009 | 3.7247 | 2.8937 | 3.3258 | 3.0709 | 2.9002 |

Supplementary Table 2. Standard deviations of absolute power of theta activity at all intervals for the control group.

| **Duration**  **Channel** | **Baseline** | **5**  **minutes** | **10**  **minutes** | **15**  **minutes** | **20**  **minutes** | **25**  **minutes** | **30**  **minutes** | **Post-**  **stimulus** |
| --- | --- | --- | --- | --- | --- | --- | --- | --- |
| Fp1 | 4.6233 | 2.0982 | 2.1920 | 2.3508 | 1.7737 | 1.8807 | 1.7628 | 1.7050 |
| F3 | 2.5190 | 2.5841 | 2.4018 | 2.5044 | 2.1642 | 2.3258 | 2.4675 | 2.7025 |
| C3 | 2.2663 | 2.6482 | 2.6370 | 2.6783 | 2.4372 | 2.5292 | 2.8989 | 3.0450 |
| P3 | 2.1972 | 2.7227 | 2.9595 | 3.2269 | 2.7137 | 3.2087 | 3.4218 | 3.4190 |
| O1 | 2.0622 | 2.9167 | 3.2245 | 3.0993 | 3.1092 | 3.4406 | 3.6461 | 3.5909 |
| F7 | 1.7870 | 1.4284 | 1.4200 | 1.3335 | 1.3113 | 1.0410 | 1.2308 | 1.3638 |
| T3 | 1.4393 | 1.4817 | 1.4287 | 1.3697 | 1.4051 | 1.2723 | 1.3691 | 1.3003 |
| T5 | 2.2052 | 2.2932 | 2.5816 | 2.8626 | 2.7928 | 2.3551 | 2.8529 | 2.4980 |
| Fz | 3.7792 | 3.4100 | 3.6409 | 3.3469 | 2.9947 | 3.3281 | 3.3665 | 3.8492 |
| Fp2 | 2.0582 | 2.0884 | 2.3038 | 2.7219 | 2.0432 | 2.0953 | 1.8934 | 2.0219 |
| F4 | 2.8087 | 2.8848 | 3.0640 | 2.7671 | 2.6270 | 2.7351 | 2.8992 | 3.2684 |
| C4 | 2.3485 | 2.5644 | 2.8626 | 2.6066 | 2.4169 | 2.6570 | 2.8155 | 3.1653 |
| P4 | 2.1525 | 2.6401 | 3.0414 | 2.6200 | 2.5560 | 2.7460 | 3.0892 | 3.3559 |
| O2 | 1.7406 | 2.6274 | 3.3736 | 3.0482 | 2.8183 | 3.1276 | 3.3630 | 3.4798 |
| F8 | 2.2055 | 1.8330 | 1.8691 | 2.3322 | 2.1491 | 1.5285 | 1.2672 | 1.5456 |
| T4 | 1.4897 | 1.2494 | 1.4894 | 1.2118 | 1.1877 | 1.1318 | 1.1652 | 1.1683 |
| T6 | 1.3518 | 1.6955 | 2.1663 | 1.7043 | 1.9055 | 1.8247 | 2.0127 | 2.1734 |
| Cz | 3.0276 | 3.0291 | 3.3702 | 3.4824 | 2.8622 | 3.1497 | 3.4526 | 3.8672 |
| Pz | 2.5213 | 2.9247 | 3.6437 | 3.5364 | 2.8674 | 3.3262 | 3.7960 | 4.0813 |

Supplementary Table 3. Standard deviations of the mean difference ($\bar{d}$) of theta activity at all intervals compared to baseline for the experimental group.

| **Duration**  **Channel** | **5**  **minutes** | **10**  **minutes** | **15**  **minutes** | **20**  **minutes** | **25**  **minutes** | **30**  **minutes** | **Post-**  **stimulus** |
| --- | --- | --- | --- | --- | --- | --- | --- |
| Fp1 | 1.6937 | 1.3969 | 1.2443 | 1.4928 | 1.0778 | 1.5940 | 1.4975 |
| F3 | 2.0293 | 1.7809 | 1.6118 | 1.3933 | 1.9215 | 2.1720 | 1.6025 |
| C3 | 1.3206 | 1.8143 | 1.7853 | 1.6503 | 1.9039 | 1.7001 | 1.7777 |
| P3 | 1.4827 | 1.6491 | 1.8335 | 1.8163 | 2.1176 | 1.2988 | 1.9602 |
| O1 | 1.4172 | 1.5079 | 1.8421 | 1.5093 | 2.1118 | 1.7173 | 1.7414 |
| F7 | 1.0304 | 1.2081 | 0.9026 | 0.8968 | 1.1480 | 1.1959 | 0.8624 |
| T3 | 0.7324 | 1.0899 | 0.6280 | 0.7676 | 1.0242 | 1.1702 | 0.9193 |
| T5 | 1.0258 | 1.6750 | 1.6591 | 1.2355 | 1.9541 | 1.2469 | 1.4741 |
| Fz | 2.4788 | 2.1602 | 2.1282 | 1.8994 | 2.2612 | 2.6477 | 1.9697 |
| Fp2 | 1.6759 | 1.5947 | 1.3073 | 1.6200 | 1.3770 | 1.6552 | 1.4341 |
| F4 | 1.9137 | 1.9132 | 2.0045 | 1.5002 | 1.9138 | 2.2580 | 1.9422 |
| C4 | 1.3430 | 1.4541 | 1.8063 | 1.5279 | 1.6093 | 1.5675 | 1.8115 |
| P4 | 1.5881 | 1.4273 | 1.7937 | 1.6472 | 1.9211 | 1.2579 | 1.8522 |
| O2 | 1.6892 | 1.2904 | 1.7429 | 2.2161 | 1.8593 | 1.5341 | 2.2007 |
| F8 | 0.9365 | 0.9190 | 0.9091 | 1.2973 | 1.2564 | 1.0787 | 1.2701 |
| T4 | 0.8145 | 0.8714 | 0.9134 | 0.8910 | 1.0253 | 1.0658 | 1.2509 |
| T6 | 0.9296 | 1.0731 | 1.1197 | 1.0978 | 1.2059 | 1.2521 | 1.4367 |
| Cz | 2.3504 | 2.5943 | 2.9004 | 2.6617 | 2.3898 | 2.2913 | 2.7826 |
| Pz | 2.1531 | 2.0133 | 2.3743 | 2.0798 | 2.4065 | 1.5745 | 2.2417 |

Supplementary Table 4. Standard deviations of the mean difference ($\bar{d}$) of theta activity at all intervals compared to baseline for the control group.

| **Duration**  **Channel** | **5**  **minutes** | **10**  **minutes** | **15**  **minutes** | **20**  **minutes** | **25**  **minutes** | **30**  **minutes** | **Post-**  **stimulus** |
| --- | --- | --- | --- | --- | --- | --- | --- |
| Fp1 | 3.3764 | 3.5553 | 3.8558 | 3.7244 | 4.2333 | 4.5240 | 4.6670 |
| F3 | 0.6654 | 1.0678 | 1.0475 | 1.1911 | 1.0111 | 1.2522 | 1.4480 |
| C3 | 0.7824 | 0.9044 | 0.8681 | 0.7902 | 1.2039 | 1.5789 | 1.5151 |
| P3 | 0.8556 | 1.2610 | 1.3264 | 0.9047 | 1.6320 | 1.7525 | 1.5278 |
| O1 | 1.1028 | 1.3923 | 1.1671 | 1.3322 | 1.8093 | 2.0560 | 1.9149 |
| F7 | 0.7767 | 1.1070 | 1.1609 | 1.2991 | 0.8963 | 1.0507 | 0.9980 |
| T3 | 0.4110 | 0.6599 | 0.7536 | 0.8330 | 0.9655 | 1.1195 | 1.0122 |
| T5 | 0.6083 | 0.8558 | 0.8901 | 0.7299 | 1.0905 | 0.9415 | 0.9558 |
| Fz | 1.2958 | 2.1060 | 2.0337 | 1.9477 | 1.9648 | 2.2594 | 2.3632 |
| Fp2 | 0.9767 | 1.3328 | 1.4365 | 0.9999 | 0.8827 | 1.2260 | 1.8381 |
| F4 | 0.9241 | 1.7751 | 1.3933 | 1.4815 | 1.6458 | 2.1070 | 2.0847 |
| C4 | 0.6649 | 1.2460 | 0.8784 | 0.9570 | 1.3636 | 1.4782 | 1.5798 |
| P4 | 1.0803 | 1.5897 | 1.1112 | 1.2554 | 1.6219 | 1.9264 | 1.9678 |
| O2 | 1.1458 | 1.8493 | 1.6313 | 1.3576 | 1.8117 | 2.0446 | 2.1432 |
| F8 | 1.2928 | 1.7623 | 1.9637 | 1.9830 | 1.5835 | 1.9376 | 1.9457 |
| T4 | 0.6610 | 1.2282 | 0.8619 | 0.9477 | 0.9513 | 1.1757 | 1.1082 |
| T6 | 0.7664 | 1.4419 | 0.8499 | 1.0018 | 1.0143 | 1.2521 | 1.4016 |
| Cz | 1.0140 | 1.3537 | 1.2396 | 1.2149 | 1.8275 | 2.0385 | 2.0117 |
| Pz | 1.1914 | 1.8484 | 1.3906 | 1.1858 | 1.9639 | 2.4187 | 2.0403 |
